# Characterizing the transcutaneous electrical recruitment of lower leg afferents in healthy adults: implications for non-invasive treatment of overactive bladder

**DOI:** 10.1186/s12894-018-0322-y

**Published:** 2018-02-13

**Authors:** Eshani Sharan, Kelly Hunter, Magdy Hassouna, Paul B. Yoo

**Affiliations:** 1 Institute of Biomaterials and Biomedical Engineering, University of Toronto, 164 College Street, Room 407, Toronto, ON M5S 3G9 Canada; 2 Department of Electrical and Computer Engineering, University of Toronto, Toronto, ON Canada; 3 Division of Urology, Toronto Western Hospital, Toronto, ON Canada

**Keywords:** Transcutaneous electrical nerve stimulation, Bladder neuromodulation, Overactive bladder, Tibial nerve, Saphenous nerve, Plantar nerve

## Abstract

**Background:**

As a potential new treatment for overactive bladder (OAB), we investigated the feasibility of non-invasively activating multiple nerve targets in the lower leg.

**Methods:**

In healthy participants, surface electrical stimulation (frequency = 20 Hz, pulse width = 200 μs) was used to target the tibial nerve, saphenous nerve, medial plantar nerve, and lateral plantar nerve. At each location, the stimulation amplitude was increased to define the thresholds for evoking (1) cutaneous sensation, (2) target nerve recruitment and (3) maximum tolerance.

**Results:**

All participants were able to tolerate stimulation amplitudes that were 2.1 ± 0.2 (range = 2.0 to 2.4) times the threshold for activating the target nerve.

**Conclusions:**

Non-invasive electrical stimulation can activate neural targets at levels that are consistent with evoking bladder-inhibitory reflex mechanisms. Further work is needed to test the clinical effects of stimulating one or more neural targets in OAB patients.

**Electronic supplementary material:**

The online version of this article (10.1186/s12894-018-0322-y) contains supplementary material, which is available to authorized users.

## Background

Overactive bladder (OAB) is characterized by symptoms of frequency and urgency that may or may not result in urinary incontinence [1]. Unlike cases of neurogenic bladder with known etiology (e.g., spinal cord injury or multiple sclerosis), OAB is commonly diagnosed in otherwise healthy individuals. OAB can affect up to 16% of adults and can adversely affect an individual’s ability to perform everyday tasks, social interactions, and sleeping habits, all of which can be quantified by significant decreases in quality of life measures [2, 3]. Widely accepted clinical therapies include behavioural modification [4], drugs, intravesical Botox [5], and sacral neuromodulation [6]. However, long-term therapeutic efficacy can be limited. Up to 80% of patients discontinue drugs within the first 6 months [7], Botox injection can cause urinary retention, and sacral neuromodulation is an expensive and relatively invasive treatment option.

As an alternative, patients may be provided with percutaneous tibial nerve stimulation (PTNS) therapy, which is a procedure performed in a clinical setting. It involves the insertion of a 34G stainless needle above the medial malleolus, through which electrical pulses are used to stimulate the tibial nerve. Nerve activation is confirmed by movement of the toes or a sensation radiating along the sole of the foot. Weekly clinical visits are repeated for 3 months, and if the patient responds to treatment, maintenance PTNS is provided every 3 weeks thereafter [8, 9].

Studies suggest that transcutaneous electrical nerve stimulation (TENS) of the tibial nerve (TN) could also provide an effective means of treating OAB. It is non-invasive, relatively inexpensive, and could allow patients to self-administer treatment at home. Since the first clinical report on the therapeutic effects of TN stimulation [10], multiple studies have demonstrated the feasibility of using TENS in OAB patients. For example, Ammi et al. showed that daily TENS applied near the ankle can significantly improve the quality of life in patients [11], and Manriquez et al. demonstrated that TENS applied twice a week can achieve clinical efficacy that is comparable to bladder medication [12]. Despite these findings, optimal therapeutic use of TENS remains unclear [13].

As a potential solution for improving the clinical efficacy, we have begun to investigate the feasibility of using TENS to electrically activate multiple nerve targets. Bladder-inhibitory responses evoked by TN stimulation is a well-documented phenomenon that has been demonstrated in anesthetized animals [14–16], and also in human participants subjected to surface stimulation of the plantar surface of the foot [17, 18]. It has also been shown in anesthetized rats that electrical stimulation of the medial and lateral plantar nerves can evoke reflexes that (1) inhibit the bladder during electrical stimulation (i.e. acute effect) or (2) cause bladder inhibition that is sustained even after the stimulus has been turned off (i.e., prolonged effect), respectively [19]. In addition, there is evidence that electrical stimulation of the saphenous nerve (SAFN) can also modulate bladder function. By implanting a nerve cuff electrode around the SAFN (immediately below the knee) in anesthetized rats [20], we showed that prolonged bladder-inhibitory responses can be evoked in a frequency-dependent manner (10 Hz to 20 Hz). And in a small cohort of OAB patients [21], we have observed significant improvements in both OAB symptoms and quality of life measures following 12 weeks of percutaneous SAFN stimulation.

In this study, we investigated the feasibility of using TENS to selectively activate multiple nerve targets located in the lower leg (tibial nerve, saphenous nerve, medial plantar nerve, and lateral plantar nerve). A commercially available TENS device was used to electrically stimulate each target in healthy participants. The primary goal was to systematically characterise the electrical recruitment of each target nerve by defining: (1) the threshold amplitude for eliciting cutaneous sensation, (2) the threshold amplitude for activating the target nerve, and (3) the maximum stimulation amplitude tolerated by participants.

## Methods

In accordance with the protocol approved by the research ethics board (REB, Approval #32461) of the University of Toronto, the study was conducted in 15 healthy participants (10 female, age = 23.9 ± 2.5 years, range = 19–28 years) who provided written consent prior to beginning each experiment. Participants were recruited using posters placed around the university campus, and e-mails sent to the students and faculty at the University of Toronto.

**Fig. 1:**
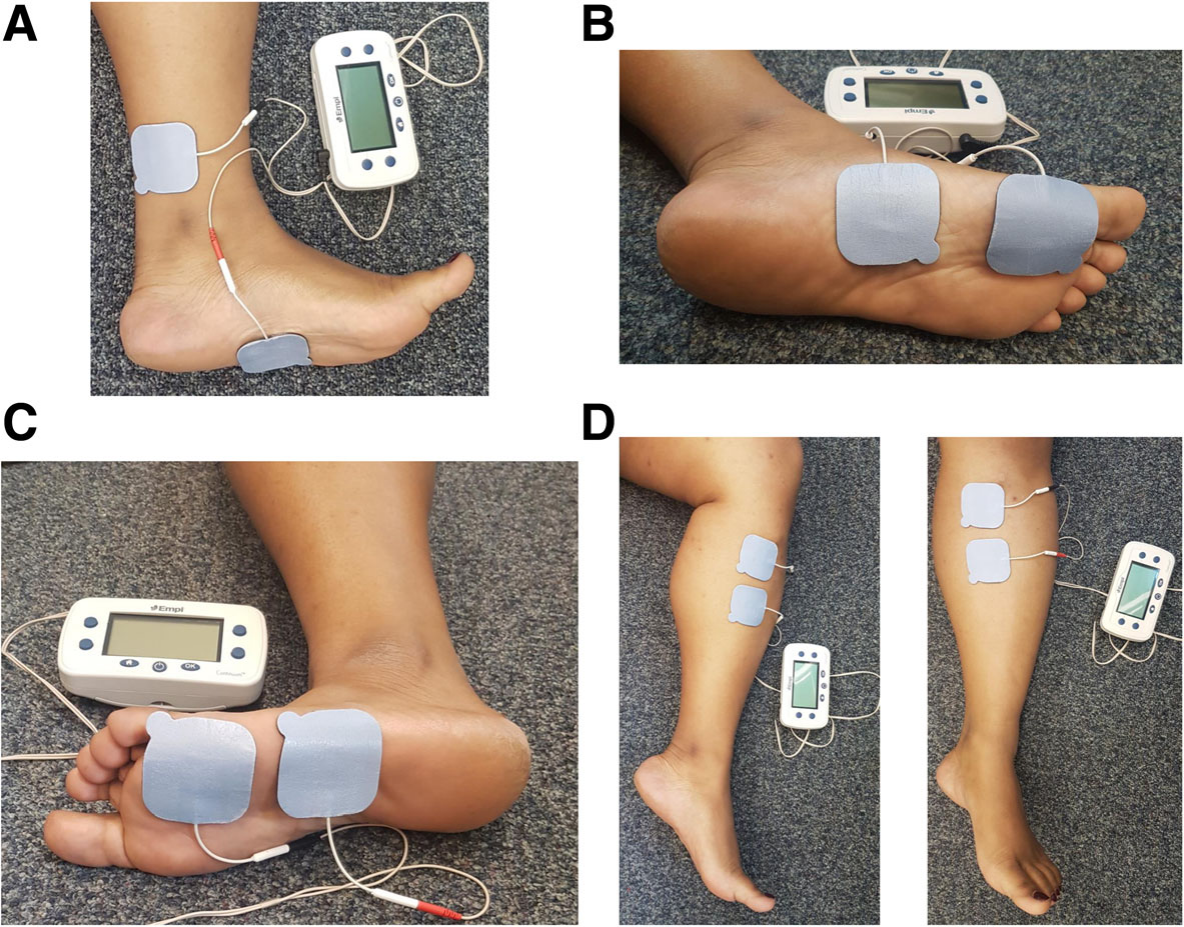
Captured images of surface electrodes used to target the 4 neural targets. **a** The tibial nerve (TN) configuration involved the cathode being placed 3 finger widths above and 1 finger width posterior to the medial malleolus, and the anode placed at the midsole of the foot. **b** The medial plantar nerve (MPN) was targeted by placing both electrodes along the medial side of the plantar foot surface: the cathode is placed at the base of the hallux and the anode is placed 2 finger widths from the cathode. **c** The lateral plantar nerve (LPN) was targeted by placing both electrodes along the lateral side of the foot. **d** The saphenous nerve (SAFN) configuration involved positioning the cathode 2 finger widths below the medial condyle of the tibia, and the anode 2 fingers widths inferior to the cathode

The experiment involved a one-hour session, during which a total of 11 stimulation trials were conducted. Following skin sterilization with alcohol wipes, a pair of 5 cm × 5 cm self-adhesive surface electrodes (STIMCARE, DJO Global, Vista, California) were placed on the lower leg to activate different neural targets (Fig. 1): tibial nerve (TN), medial plantar nerve (MPN), the lateral plantar nerve (LPN), and the saphenous nerve (SAFN). Both electrodes were connected to a hand-held TENS unit (Empi Continuum™, DJO Global, Vista, California), where the stimulation frequency (20 Hz) and pulse width (200 μs) were set at constant values.

The electrical activation of each neural target involved a series of 3 stimulation trials, where the amplitude was increased from 0 mA up to a pre-defined endpoint. The first trial was terminated at the cutaneous sensory threshold, where the participant felt the stimulation at the surface electrode (T_skin_). The second trial was terminated at the threshold for activating the target nerve (T_nerve_), where the participant for example began to feel paresthesia radiate distally along the medial surface of the leg. Finally, the third trial was terminated when the participant could no longer tolerate TENS stimulation (T_limit_). The 4 neural targets were tested in randomized order by conducting a set of stimulation trials on one leg (e.g., TN + right leg) and then switching to the contralateral leg to test another nerve (e.g., MPN + left leg). Any potential carry-over effects of stimulation were minimized by alternating neural targets between each leg. The nerve activation threshold (T_nerve_) was confirmed by either a foot motor response (TN, LPN, and MPN) or a cutaneous sensation that radiated down the medial aspect of the lower leg (SAFN). Immediately following each trial, the participant was provided a questionnaire that asked the individual to quantify the perceived intensity of surface stimulation using a visual analogue scale (VAS range = 1 to 5), where 1 indicated the least comfortable sensation (Additional file 1). The questionnaire also instructed each participant to indicate the perceived area of stimulation by shading in an anatomical grid of the lower leg. All raw data involving nerve activation thresholds and VAS scores are provided in this manuscript (Additional file 2).

### Data analysis

**Fig. 2:**
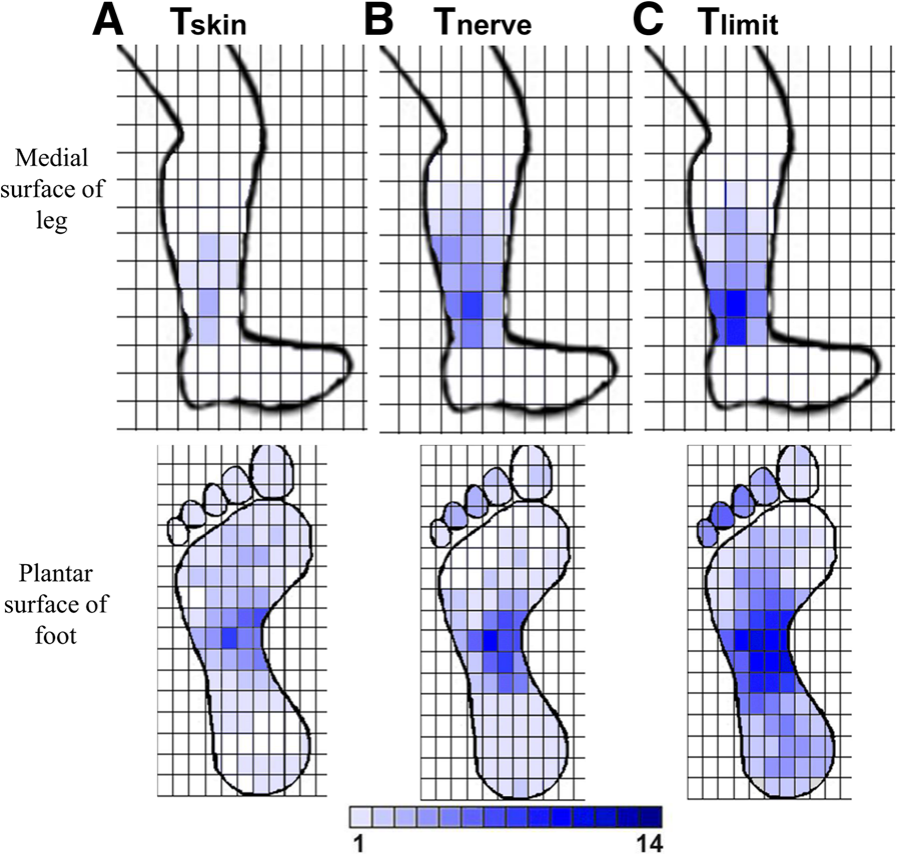
Shaded anatomical plot of the sensation perceived during TN stimulation. The color of each square within the grid represents the cumulative number of participants that felt stimulation at that location. Progressive spread of the perceived sensation occurred as the stimulation amplitude was increased: (**a**) T_skin_, (**b**) T_nerve_, and (**c**) T_limit_. There were increases in the ‘activated’ areas both on the plantar foot surface and the medial aspect of the lower leg. It is noted that the color scale (1 to 14) indicates that not all 15 participants shaded in the same pixels in response to stimulation. (same as in Fig. 3)

The stimulation amplitudes that achieved threshold activation of the skin (T_skin_), target nerve (T_nerve_), and maximum tolerance (T_limit_) were summarized across all participants and represented as the mean ± standard deviation. Since each participant exhibited different cutaneous thresholds, the T_nerve_ and T_limit_ values for each nerve target were normalized to the participant’s T_skin_. Data obtained from the questionnaire was used to summarize the perceived intensity of stimulation (VAS scores), and to also generate anatomical plots that show the spatial distribution of stimulation-evoked ‘sensation’. An anatomical plot for each neural target was created by summing the total number of participants that shaded in a particular pixel within the grid (maximum = 15), and then assigning a color intensity that was proportional to the number of participants who perceived stimulation in that particular pixel (Fig. 2). Statistical analysis was conducted by performing a one-way ANOVA followed by a pair-wise Tukey-Kramer multi-comparisons (JMP, SAS Institute Inc.©, Cary, NC). A *p*-value less than 0.05 was considered statistically significant.

## Results

**Fig. 3:**
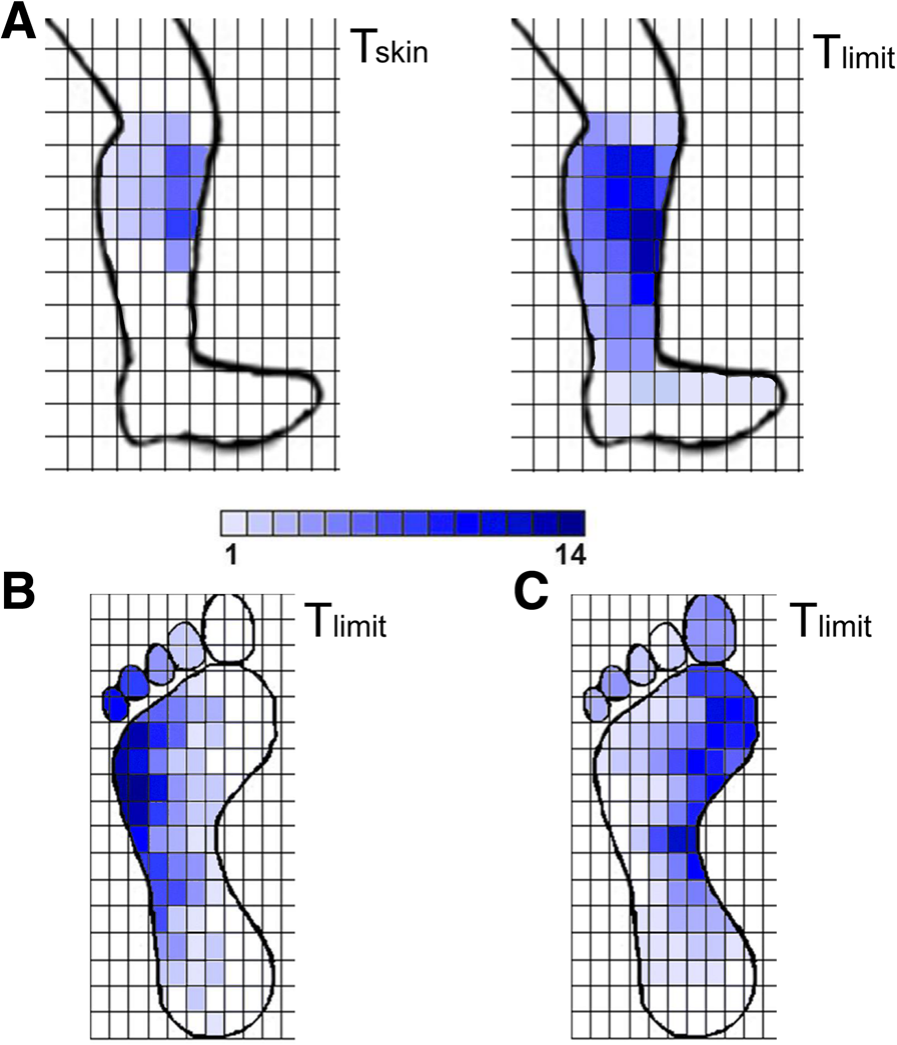
Shaded anatomical plot of the sensation perceived during (**a**) SAFN, (B) LPN, and (**c**) MPN. As the amplitude was increased during SAFN stimulation from T_skin_ to T_limit_, the evoked sensation spread across the entire medial aspect of the lower leg, down to the ankle. As shown for LPN and MPN stimulation, the perceived area of sensation at T_limit_ was generally consistent with the innervation pattern of the lateral and plantar nerves, respectively

Transcutaneous electrical activation of the 4 neural targets (TN, SAFN, MPN, and LPN) was achieved in all 15 healthy participants. Each participant was able to indicate graphically the anatomical representation of electrical stimulation that was perceived at T_skin_ and T_limit_. As shown in Fig. 2, the perceived sensation of electrical pulses applied at T_skin_ was spatially limited to the location of the surface electrodes. At T_limit_, the anatomical plots show notable spread of sensation radiating away from the surface electrodes. Participants receiving TN stimulation indicated the evoked sensation spread up the medial aspect of the lower leg and also across a larger area of the ventral foot surface. Participants receiving SAFN stimulation indicated that the evoked sensation consistently radiated down to the medial malleolus (Fig. 3a). In contrast, electrical stimulation of the MPN and LPN at T_limit_ resulted in perceived ‘sensations’ that were consistent with selective nerve activation (i.e., minimal spillover into adjacent innervation area, Fig. 3b-c).

As shown in Table 1, the average stimulation amplitude needed to evoke a cutaneous sensation using any of the 4 configurations ranged from 8.7 mA to 13.6 mA. The SAFN configuration exhibited the lowest T_skin_, which was significantly lower than T_skin_ for MPN and LPN stimulation (*p* < 0.05). The stimulation amplitude required to activate the underlying target nerve (T_nerve_) increased substantially from T_skin_, where the amplitude needed to activate the TN was found to be significantly lower than that for SAFN and LPN activation (p < 0.05). The average stimulation amplitude at which maximum tolerance was achieved (T_limit_) ranged between 42.2 mA to 50.2 mA, with no statistical difference among the four targets.

**Table 1:** Summary of TENS activation thresholds: mean ± SD (range)

Target Nerve	T_skin_ (mA)	T_nerve_ (mA)	T_limit_ (mA)
TN	10.2 ± 2.8 (6–17)	19.7 ± 4.4 (9–30)	42.2 ± 2.5 (22–64)
SAFN	8.7 ± 2.3 (6–15)	25.7 ± 7.4 (17–41)	47.7 ± 9.3 (28–62)
LPN	13.6 ± 3.7 (6–22)	25.5 ± 6.1 (19–41)	50.2 ± 15.5 (28–85)
MPN	11.6 ± 4.2 (3.5–19)	21.7 ± 5.9 (15–39)	50.1 ± 23.0 (25–100)

**Fig. 4:**
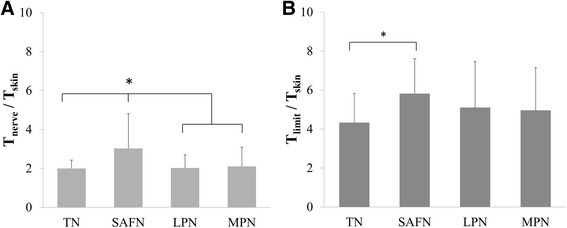
Comparison of (**a**) T_nerve_ and (**b**) T_limit_ values, which were both normalized to the cutaneous activation threshold (T_skin_). **a** The relatively larger normalized T_nerve_ for SAFN activation (3.0 ± 1.8) suggests greater activation of cutaneous afferents before electrical recruitment of the SAFN branches. **b** In contrast, SAFN stimulation can be applied at comparatively higher multiples of T_skin_ (5.8 ± 1.8) before participants reach maximum tolerance. [*, *p* < 0.05]

**Fig. 5:**
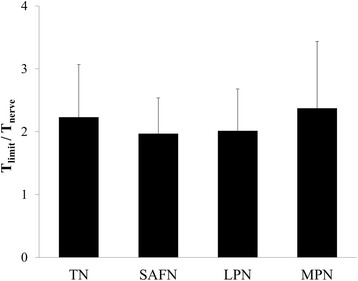
Transcutaneous recruitment of the target nerve was expressed in relation to the maximum tolerable stimulation amplitude reported by each participant. The range of normalized T_nerve_ values was consistent across all 4 stimulation configurations: 2.1 ± 0.2 (range = 2.0 to 2.4). Statistically, there was no significant difference among the different neural targets (*p* = 0.48)

**Fig. 6:**
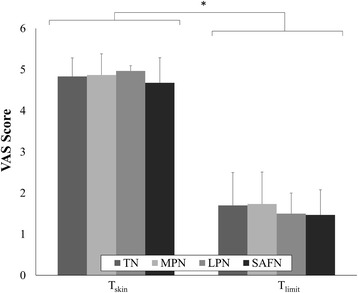
The sensations evoked by transcutaneous stimulation were quantified with visual analogue score (VAS) measurements taken at T_skin_ and T_limit_. Regardless of the stimulation configuration, the average VAS scores were consistent with cutaneous activation and maximum tolerance: TN (4.8 ± 0.4, 1.7 ± 0.8), MPN (4.9 ± 0.5, 1.7 ± 0.8), LPN (5.0 ± 0.1, 1.5 ± 0.5), SAFN (4.7 ± 0.6, 1.5 ± 0.6), respectively. [*, *p* < 0.05]

When comparing T_nerve_ and T_limit_ (both normalized with respect to T_skin_), we found that the SAFN configuration required larger amplitudes to recruit the underlying nerve bundle (Fig. 4a), and to also reach maximum tolerance (Fig. 4b). As indicated in Table 1, these findings were primarily due to the significantly lower T_skin_ achieved by TENS activation of the SAFN. However, regardless of the stimulation configuration, TENS was able to provide electrical stimulation up to 2.1 ± 0.2 (range = 2.0 to 2.4) times the nerve activation threshold (Fig. 5). The summarized VAS scores quantitatively confirmed the significantly larger sensations (e.g. lower VAS scores) perceived by participants when TENS was applied at T_limit_ relative to T_skin_ (Fig. 6). The perceived level of intensity was similar across the different stimulation sites.

## Discussion

In this study, we characterized the electrical activation of 4 different nerve targets which may be considered for providing non-invasive treatment of OAB. In healthy adult subjects, we found that selective activation of the MPN, LPN and SAFN was possible up to approximately 2.1 times the target nerve activation threshold (T_nerve_). Similar to our previous computational study of PTNS [22], co-activation of SAFN fibers was observed in every participant when TENS was used to target the TN trunk. The level of target nerve activation that was achieved in this study are consistent with the stimulation amplitudes required to evoke bladder-inhibitory responses in anesthetized animals [16, 23], and also for achieving therapeutic effects with PTNS [8, 9, 24]. It was recently shown clinically that both plantar nerves can be simultaneously stimulated to elicit bladder-inhibitory responses [17, 18, 25].

Multiple studies show that transcutaneous TN stimulation can elicit therapeutic effects in OAB patients. One of the most common electrode configurations used to stimulate the TN involves one electrode being placed immediately posterior to the medial malleolus and the return electrode placed 5 cm to 10 cm cephalad to the first [11, 26, 27]. In these studies, the authors reported treatment success rates ranging between 53% and 87% of patients. Patidar et al. used a different TN configuration (one electrode just above the medial malleolus and the second electrode placed 5 cm cephalad to the first) to achieve a comparable 71% response rate in a group of pediatric OAB patients [28]. Whereas, other researchers achieved TN stimulation by placing surface electrodes in similar fashion to PTNS (one above the medial malleolus and the other below) [12, 29].

The anatomical sensory maps generated by TN stimulation in this study (Fig. 2) indicate that co-activation of subsets of SAFN fibers occurs at and above the TN activation threshold (T_nerve_). These results are consistent with computational simulations of PTNS [22, 30] and further support the notion that electrical activation of SAFN fibers can potentially contribute to the clinical effects of PTNS therapy. Given the disparate spinal projections of the SAFN (L2-L4) and TN (L5-S4) in humans, it is likely that both neural inputs involve different neural mechanisms. However, it is currently unclear whether co-activation of TN and SAFN afferents will have any significant effect on treating OAB symptoms, when compared to TN stimulation alone.

Non-invasive TENS has the advantage of being a low risk (minimal side-effects), low cost, and convenient technology that can help patients chronically manage OAB symptoms. Although limited in number, published clinical trials involving TENS of the TN demonstrate therapeutic response rates that are relatively consistent with those achieved by PTNS [31]. However, optimization of long-term TENS therapies will require further work in determining the proper electrode size that will enable maximum tolerable stimulation amplitudes [32], allowing patients to find their most effective or comfortable set of stimulation parameters (e.g, frequency), and also assessing different types of clinical support (e.g., urology clinic, physiotherapy clinic) that will maximize patient compliance.

## Conclusion

This study shows the feasibility of non-invasively stimulating neural targets in the lower leg that are aimed at treating OAB. In healthy adult subjects, we confirmed that each targeted nerve (TN, SAFN, MPN, LPN) achieved activation levels that are relevant to evoking bladder-inhibitory reflexes. Further clinical studies are necessary to determine whether individual or co-activation of these neural targets with TENS can be used to treat OAB patients.

## Additional files

Additional file 1: Questionnaire.doc. Visual analog scale and anatomical maps. This questionnaire was provided to each participant to quantitatively measure the sensation of TENS and the physical spread of stimulation-evoked sensation as the amplitude was increased. (DOCX 357 kb)

Additional file 2: Raw Data.xlsx. Summary of raw data obtained from each participant that characterized the stimulation threshold values (Raw Data tab) and the visual analogue scale values (Comfort Ratings tab). (XLSX 17 kb)

## Abbreviations

LPN: Lateral plantar nerve
MPN: Medial plantar nerve
OAB: Overactive bladder
PTNS: Percutaneous tibial nerve stimulation
SAFN: Saphenous nerve
TENS: Transcutaneous tibial nerve stimulation
TN: Tibial nerve
VAS: Visual analog scale
