# Supplementary figures and images for: Characterizing the transcutaneous electrical recruitment of lower leg afferents in healthy adults: implications for non-invasive treatment of overactive bladder
Source: BMC Urol. 2018 Feb 13;18:10. doi: 10.1186/s12894-018-0322-y (PMC5812114; doi:10.1186/s12894-018-0322-y)

## Questionnaire of TENS Study


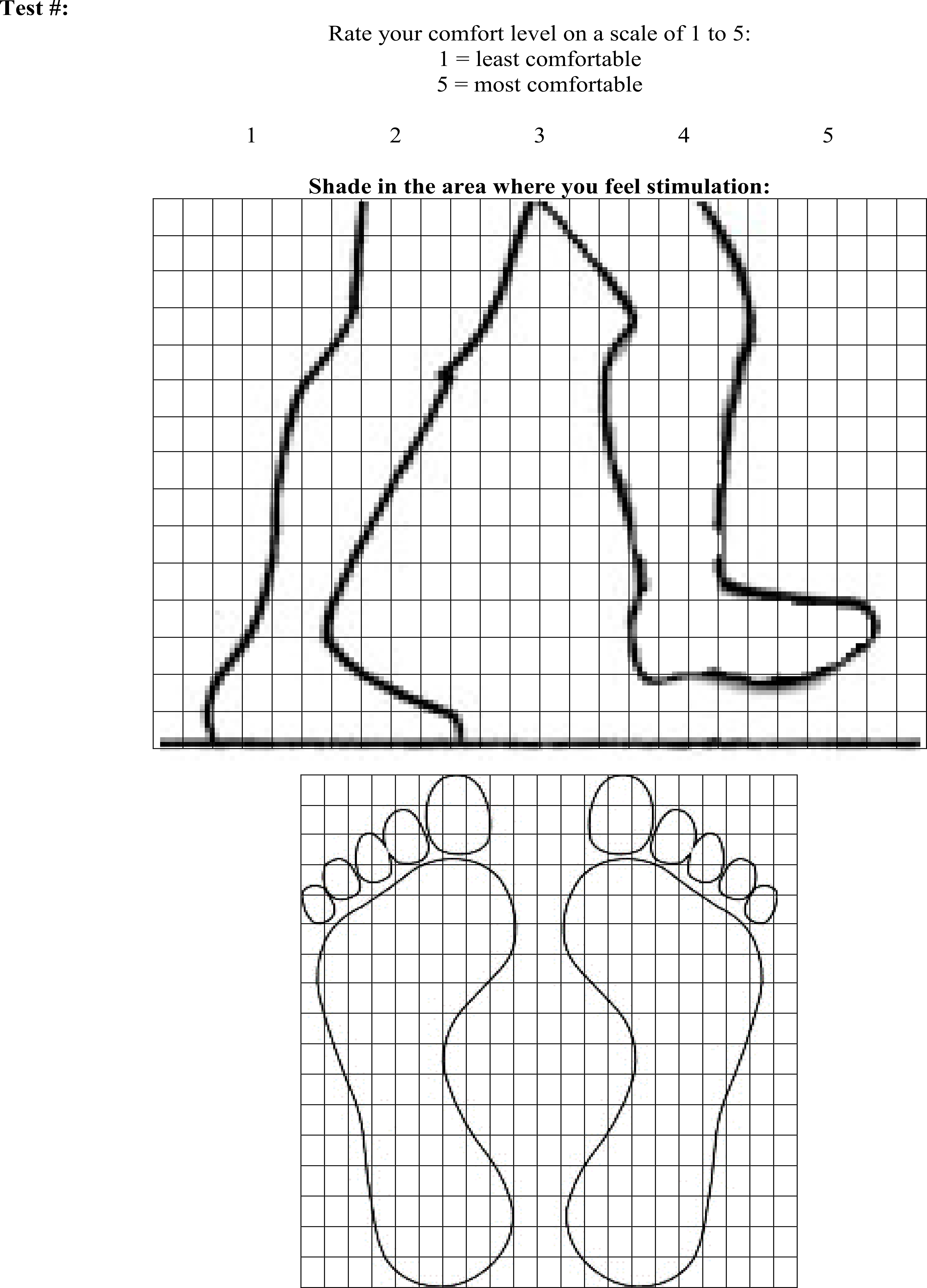

Supplement: Supplementary file 1 — Questionnaire.doc. Visual analog scale and anatomical maps. This questionnaire was provided to each participant to quantitatively measure the sensation of TENS and the physical spread of stimulation-evoked sensation as the amplitude was increased. (DOCX 357 kb) [file 12894_2018_322_MOESM1_ESM.docx]
